# ICR142 Benchmarker: evaluating, optimising and benchmarking variant calling performance using the ICR142 NGS validation series

**DOI:** 10.12688/wellcomeopenres.14754.2

**Published:** 2018-10-31

**Authors:** Elise Ruark, Esty Holt, Anthony Renwick, Márton Münz, Matthew Wakeling, Sian Ellard, Shazia Mahamdallie, Shawn Yost, Nazneen Rahman

**Affiliations:** 1 Division of Genetics & Epidemiology, The Institute of Cancer Research, London, SM2 5NG, UK; 2 Institute of Biomedical and Clinical Science, University of Exeter Medical School, Exeter, EX2 5DW, UK; 3 Cancer Genetics Unit, Royal Marsden NHS Foundation Trust, London, SM2 5PT, UK

**Keywords:** Variant calling, next generation sequencing, benchmarking, specificity, sensitivity, false detection rate, GATK, OpEx, DeepVariant

## Abstract

Evaluating, optimising and benchmarking of next generation sequencing (NGS) variant calling performance are essential requirements for clinical, commercial and academic NGS pipelines. Such assessments should be performed in a consistent, transparent and reproducible fashion, using independently, orthogonally generated data.

Here we present ICR142 Benchmarker, a tool to generate outputs for assessing germline base substitution and indel calling performance using the ICR142 NGS validation series, a dataset of Illumina platform-based exome sequence data from 142 samples together with Sanger sequence data at 704 sites. ICR142 Benchmarker provides summary and detailed information on the sensitivity, specificity and false detection rates of variant callers. ICR142 Benchmarker also automatically generates a single page report highlighting key performance metrics and how performance compares to widely-used open-source tools.

We used ICR142 Benchmarker with VCF files outputted by GATK, OpEx and DeepVariant to create a benchmark for variant calling performance. This evaluation revealed pipeline-specific differences and shared challenges in variant calling, for example in detecting indels in short repeating sequence motifs. We next used ICR142 Benchmarker to perform regression testing with DeepVariant versions 0.5.2 and 0.6.1. This showed that v0.6.1 improves variant calling performance, but there was evidence of minor changes in indel calling behaviour that may benefit from attention. The data also allowed us to evaluate filters to optimise DeepVariant calling, and we recommend using 30 as the QUAL threshold for base substitution calls when using DeepVariant v0.6.1.

Finally, we used ICR142 Benchmarker with VCF files from two commercial variant calling providers to facilitate optimisation of their in-house pipelines and to provide transparent benchmarking of their performance.

ICR142 Benchmarker consistently and transparently analyses variant calling performance based on the ICR142 NGS validation series, using the standard VCF input and outputting informative metrics to enable user understanding of pipeline performance. ICR142 Benchmarker is freely available at https://github.com/RahmanTeamDevelopment/ICR142_Benchmarker/releases.

## Introduction

Variant calling from next generation sequencing (NGS) data is a highly active area of bioinformatics, important to many clinical, commercial and academic applications. Several open-source tools are available and have been integrated into variant calling pipelines by many laboratories ^1– 6^. Commercial solutions and/or in-house proprietary tools are also increasingly being used by NGS analysis providers. Evaluations of pipeline performance are often based on internal data. This makes comparison, standardisation and regulation of NGS variant calling performance difficult ^7^.

Assessment of variant calling performance is vital for improvement and optimisation of NGS variant calling. Comparative performance across pipelines is also of increasing importance, as the number of different analysis tools and providers continues to expand. The availability of benchmarking datasets with orthogonally confirmed positive and negative sites are required for optimal independent assessment of sensitivity, specificity and false detection rates (FDR). 

We previously made available the ICR142 NGS validation series that includes NGS and Sanger data from 142 samples ^8^. To construct the ICR142 NGS validation series we analysed exome sequence data from the 142 samples with multiple variant callers and undertook Sanger sequencing analysis at 704 sites to generate a dataset useful for systematic, transparent variant calling assessment and comparison ^8^.

Here we present ICR142 Benchmarker ^9^, a tool to generate outputs for assessing variant calling performance using the ICR142 NGS validation series. We used ICR142 Benchmarker with VCF files from GATK, OpEx and DeepVariant to provide guidance on expected variant caller performance compared to three open-source pipelines ^1, 10, 11^. We then used ICR142 Benchmarker with VCF files from two commercial NGS variant calling providers, to provide comparison data to facilitate optimisation of their in-house pipelines, and to help give transparency of performance for their customers.

## Methods

### ICR142 NGS validation series

**Figure 1.**
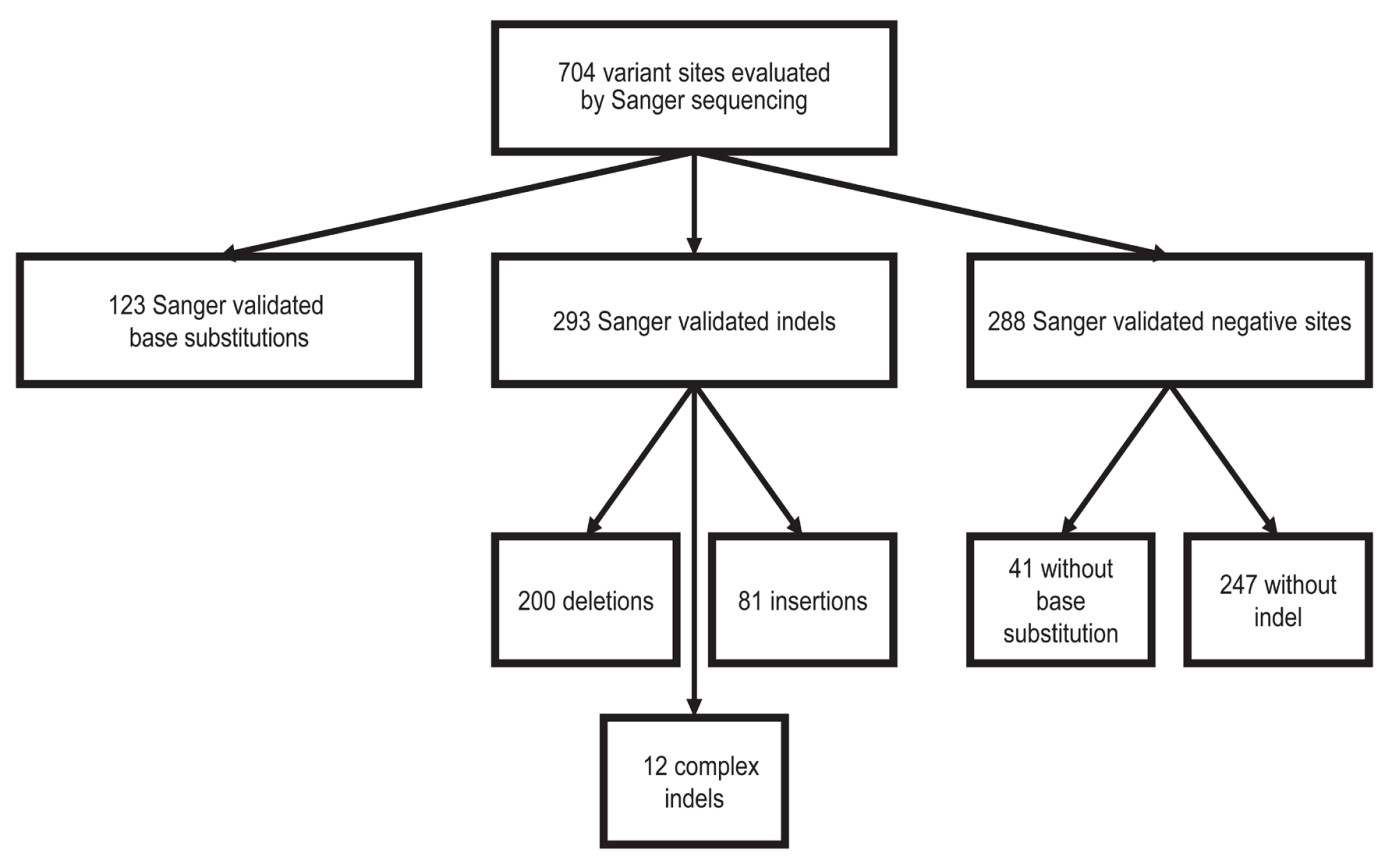
Layout of the 704 Sanger validated variant sites. Breakdown of the 704 Sanger validated base substitutions, insertion and/or deletions (indels), and negative sites from 142 samples. The diagram shows the exact number of base substitutions, deletions, insertions, complex indels, and sites without a base substitution or indel.

The ICR142 NGS validation series is a dataset that includes high-quality exome sequence data from 142 samples together with Sanger sequence data at 704 sites; 416 sites with variants and 288 sites at which variants were called by a variant caller, but no variant is present in the corresponding Sanger sequence. The exome sequence data was generated using the Illumina TruSeq Exome and a HiSeq2000 sequencer. Full details of the ICR142 series are given in Ruark *et al.* ^8^. In total, the ICR142 NGS validation series includes 704 sites, comprised of 123 base substitution variants, 293 insertions and/or deletion (indel) variants, 41 negative base substitution sites and 247 negative indel sites ( Figure 1) ^8^.

To determine if a variant was present we examined each Sanger sequence with Chromas software v2.13. For each site we selected an ENST from release 65 as the reference sequence. We analysed a region of interest of at least 100 base pairs (bp) of sequence flanking each variant site to allow for position/annotation errors.

We considered a base substitution to be confirmed if the correct variant was called at the exact position and the variant base signal was accompanied by a corresponding reduction in the reference base signal. We considered an indel variant to be confirmed if an indel variant was present in the region of interest and the indel variant allele signal was present along the complete length of the region of interest.

We considered a site negative for a base substitution if the exact base substitution was not present. We considered a site negative for an indel if no indel was detected in the 200bp region of interest.

### ICR142 Benchmarker

***Implementation.*** ICR142 Benchmarker ^9^ is implemented as an easy-to-use tool for assessing variant calling performance using the ICR142 NGS validation series. It can be used with hg19/GRCh37 or hg38/GRCh38 data. The tool includes an analysis script and three supporting files: the Sanger data file, the Report template file and the descriptive ColumnHeadings.txt file. ICR142 Benchmarker provides a series of informative metrics with increasing levels of detail from overall calling performance to per site profiles together with a one page report summarising both standalone performance and comparative performance against widely-used open-source pipelines. ICR142 Benchmarker is implemented in R and is publically available at https://github.com/RahmanTeamDevelopment/ICR142_Benchmarker/releases.

ICR142 Benchmarker requires an input file containing the paths to VCF version 4.X files. The VCF files must each represent a single sample. The script expects the ALT column to contain only one call. Any base substitution calls are expected to have REF and ALT values of length one, e.g. REF / ALT of GTCA / ATCA should be trimmed to G / A. Multi-sample VCF or gVCF files should be parsed to fulfil the above criteria.

At each site, ICR142 Benchmarker assesses both variant detection and accuracy of variant representation, with missing genotypes allowed. Base substitution variants are both detected and accurately represented if the correct variant is called at the exact position. If an incorrect base substitution is called at that position it is considered a missed variant. For negative base substitution sites a false positive base substitution call is assigned if any base substitution call is made at the exact position. Due to the more complex nature of indel detection and representation, a stringent exact matching approach is not appropriate. We thus report indel detection as the number of indel calls within a 200bp window centred on the site position, for both true indel and negative indel sites. An indel variant is considered to be both detected and accurately represented if an exact match is found. For negative indel sites a false indel call is assigned if the indel detection value is greater than 0. Summary metrics are calculated from the detection values. Any missing values are treated as ‘no call’ in the metric calculations.

ICR142 Benchmarker generates five output files, four tab-separated .txt files and one Word document .docx. The Summary.txt file provides summary performance metrics for the evaluated method, specifically the overall sensitivity, specificity and false detection rate (FDR) values. These three metrics are also separately calculated for base substitutions or indels. The FullResults.txt file contains all of the Sanger validation information from the ICR142 dataset and information on site-specific performance at each of the 704 sites. The FalsePositives.txt and TruePositives.txt files contain the relevant lines of the input VCF files for false positive and true positive variant calls, respectively. Detailed description of all columns in the .txt files is provided in the ColumnHeaders.txt supporting file.

The Report.docx file provides a summary variant calling analysis report of performance using the ICR142 dataset. This single page document is directly constructed from the Summary.txt and FullResults.txt files and thus is transparent and reproducible. Key points from the detailed outputs are highlighted to the user, including information about performance compared to widely-used open-source variant callers.

***Operation.*** ICR142 Benchmarker can be installed by running a simple Bash script. Installation requires R version 3.1.2 or later and a capacity to build packages from source. ICR142 Benchmarker implements full version control using packrat ^12^. This approach ensures ICR142 Benchmarker implementation will not be affected by future changes of incorporated packages or their dependencies. Once installed, the tool can be run from a Linux/Unix command line. The ICR142 Benchmarker documentation is available at GitHub: https://github.com/RahmanTeamDevelopment/ICR142_Benchmarker/.

### Assessing variant calling performance

To evaluate the utility of the ICR142 NGS validation series and ICR142 Benchmarker we analysed data from three different open-source variant callers, GATK, OpEx and DeepVariant ^1, 10, 11^ and two commercial variant callers from Company A and Company B.

To generate BAM files for the GATK analysis, we aligned the ICR142 FASTQ files with BWA-MEM v0.7.12 and removed duplicates using Picard v1.129 ^13^. We ran a GATK v3.4-46 analysis on the 142 BAM files to create a multi-sample VCF file ( Supplementary File 1). We then applied standard additional filters of AB > 0.2, DP ≥ 10 and GQ ≥ 20 and included the remaining variants as the GATK set.

We ran OpEx v1.0.0, which uses Platypus v0.1.5 as its variant caller, with default settings to generate 142 single sample VCF output files from the ICR142 FASTQ files ^11^. Variants flagged as “high” by OpEx were included as the OpEx set.

We ran DeepVariant ^10^ with default settings using the OpEx BAM files to generate 142 gVCF output files. Two versions of DeepVariant were run; v0.5.2 and v0.6.1.

Two commercial variant calling providers, referred to as Company A and Company B, supplied data for the ICR142 validation series, Company A supplied 142 individual VCF files and Company B supplied a multi-sample gVCF file.

We pre-processed all multi-sample and gVCF files to ensure compatibility with the script. Multi-sample files were split into 142 single sample files with the vcf-subset command in vcftools v0.1.14 ^14^. For each gVCF file, we analysed the variant call subset to generate an initial result for each site. The reference call subset was then used to assign a missing value at sites with no call.

## Results

### Variant detection performance

We used the data from GATK, OpEx and DeepVariant v0.6.1 to provide a baseline for expected variant calling performance ( Table 1 and Supplementary File 2). Comparison of the three pipelines showed concordance at 92% of sites, both positive and negative. Because the same alignment files were used for both OpEx and DeepVariant (BWA), with a related but different aligner used for GATK (BWA-MEM), one might have expected the OpEx and DeepVariant results to be more similar to one another than to GATK. However, the aligner used did not seem to have a strong impact, as GATK and DeepVariant showed similar performance, while OpEx had a better false detection rate but lower sensitivity. This was expected, as the OpEx “high” quality filter was designed to achieve exactly this balance for its first-pass exome sequence analysis ^11^.

**Table 1.** Performance of multiple variant callers based on the ICR142 dataset. Performance metrics were calculated as: Sensitivity = TP/(TP+FN), Specificity = TN/(TN+FP) or False detection rate = FP/(FP+TP), where TP = true positive sites; TN = true negative sites; FP = false positive sites; FN = false negative sites as described in Methods. The ICR142 dataset was generated using the Illumina TruSeq exome.

	Variant type	BWA + GATK	OpEx (Stampy + Platypus)	Stampy + DeepVariant
Sensitivity	Overall	404/416 (97%)	391/416 (94%)	405/416 (97%)
	Base substitutions	123/123 (100%)	118/123 (96%)	123/123 (100%)
	Indels	281/293 (96%)	273/293 (93%)	282/293 (96%)
Specificity	Overall	266/288 (92%)	279/288 (97%)	270/288 (94%)
	Base substitutions	39/41 (95%)	39/41 (95%)	35/41 (85%)
	Indels	227/247 (92%)	240/247 (97%)	235/247 (95%)
False detection rate	Overall	22/426 (5%)	9/400 (2%)	18/423 (4%)
	Base substitutions	2/125 (2%)	2/120 (2%)	6/129 (5%)
	Indels	20/301 (7%)	7/280 (2%)	12/294 (4%)

Sites where all three pipelines called false positives highlight common challenges in variant calling. False positive base substitutions in *POTEH* and *CHEK2* are likely due to non-specific capture of sequences derived from homologous genomic sequences. For example, the region surrounding the false positive position on chromosome 22 in *POTEH* has 90–95% homology with other paralogs of the *POTE* gene, with the allele at the exact position varying between them. False positive indels called in *MUC13* and *SLC39A14* provide examples of the challenges of variant calling in short repeating sequence motifs in NGS data. Although it is possible that the Sanger result is a false negative at these sites, we consider this to be unlikely.

The discordant false positive values reveal pipeline-specific differences in variant calling performance. GATK had seven unique sites with false positives, i.e. not called by either OpEx or DeepVariant, all of which were indels. These included two sites with a long insertion (SiteIDs 31, 290) and one site with a cluster of multiple calls (SiteID 75) ( Supplementary File 2). Within the cluster, there would be no overall change in length if one could assume that all calls occurred on the same allele. However, phasing information was not provided by GATK until v3.3, and is only run automatically under specific conditions in more recent versions. Users of GATK should be cautious when multiple indels are called in close proximity in the same sample and consider visual inspection of the BAM file to check phasing, if phasing was not performed automatically. DeepVariant had four unique sites with false positives, all of which were base substitutions with QUAL value ranging from 9.3–20.3 ^15^. OpEx did not have any unique sites with false positives, consistent with the priority of the high quality OpEx filter to limit the false detection rate.

### Indel detection and representation accuracy

ICR142 Benchmarker makes a distinction between variant detection and accurate variant representation since indels detected by NGS are often validated by an orthogonal technique such as Sanger sequencing, and the call amended if required. There were ten variants which were detected by all three pipelines but not correctly represented by at least one pipeline. Nine variants were incorrectly represented by all three pipelines. These were all complex indels, indicating the need for improvement or further standardisation in the representation of this important class of variant. The final variant, an inframe deletion of 24bp in *GPRIN1* (SiteID 607), was correctly represented by OpEx but both GATK and DeepVariant represented this variant as two separate frameshifting deletions of 13bp and 11bp. This is a crucial difference, as the functional impact of inframe and frameshifting variants is often markedly different. Excluding complex indels, all three methods had greater than 98% accuracy, only OpEx achieved 100% accuracy, with all of the 264 detected insertions or deletions correctly represented.

### Utility in variant calling regression testing

Variant calling pipelines are frequently updated. Regression testing ensures previously developed and tested pipelines still perform in the same way after updates have been implemented. The ICR142 NGS validation series allows independent regression testing, and we believe it could be usefully incorporated into variant calling development processes, particularly in the clinical setting.

To investigate this we performed regression testing by performing the same ICR142 Benchmarker analysis with DeepVariant v0.5.2 and v0.6.1. We found that v0.6.1 improved on v0.5.2 across all metrics, for both base substitutions and indels ^15^. Comparison of the site-specific performance allowed us to draw more detailed insights. There were ten sites with a change in performance ( Supplementary File 3). For two sites (SiteID 34, 666) with a single correctly detected indel, v0.6.1 detected an additional indel, an unexpected change in indel calling behaviour. For seven sites the calling performance improved, with one false positive base substitution and two false positive indels no longer called and four previously undetected indel variants now called by v0.6.1. However, one site had decreased performance, with three false positive indels newly called by v0.6.1 at a site in *PABPC3* (SiteID 114). As one of the four newly detected indel variants occurred at a nearby site in *PABPC3* in a different sample, this indicates that the improved calling performance comes at the cost of additional false positives at one site. Taken together, these data indicate that v0.6.1 provides validated improvement on v0.5.2, with some caveats that may inform future updates.

### Utility in creation of variant detection filters

Many variant callers apply filters of the raw calls to improve performance. We believe the ICR142 validation series and ICR142 Benchmarker can be used to inform optimal filter creation. To evaluate this we investigated the performance of DeepVariant v0.6.1. We found that while sensitivity was excellent for both base substitutions and indels, specificity was surprisingly low for base substitutions at 85% ( Table 1). We looked at the quality information returned by DeepVariant for all base substitution positive and negative sites in the ICR142 series ^15^. We found that imposing a threshold of 30 on the QUAL column for base substitution calls reduced the false detection rate from 5% to 2%, increased the specificity to 95%, and did not greatly reduce the sensitivity, as only one variant was excluded, which had a QUAL value of 29.3, resulting in a sensitivity of 99%. We thus recommend using a filter of QUAL threshold of 30 for base substitution calls when using DeepVariant v0.6.1.

### Benchmarking variant detection performance

Using the concordant data from the open-source pipelines allowed us to describe the expected baseline performance for variant calling ( Table 2 and Supplementary File 2). There were 387 variants detected by GATK, OpEx and DeepVariant, which we call Group A variants. Any method seeking to perform as well as these open-source pipelines should be able to detect all variants in Group A. There were 261 sites where no variant was detected, which we call Group B. Methods aiming to have equivalent performance to open-source pipelines should avoid making variant calls at all Group B sites. Failure to detect a Group A variant or calling a variant at a Group B site indicates substandard performance and warrants further investigation at the algorithmic and/or filtering stage.

**Table 2.** Expected baseline performance for variant calling. Group A – variants that should be detected by any variant calling pipeline; Group B – sites in which a base substitution or insertion and/or deletion should not be called.

	Number of Sites
**Group A**	
- Base substitution variants	118
- Deletion variants	186
- Insertion variants	74
- Complex indel variants	9
**Group B**	
- No base substitution	35
- No indel	226

To demonstrate the utility of ICR142 Benchmarker to provide useful comparative performance information, we assessed variant calling by two commercial pipelines, which we call Company A and Company B ( Supplementary File 4 and Supplementary File 5).

Company A showed overall good sensitivity (96%), specificity (95%) and false detection rate (4%) ( Supplementary File 4). However, six Group A variants were not called and a false positive was called at one Group B site. The lower than typical ability to detect Group A variants indicates further work should be performed to understand why these variants were missed.

Company B also showed overall good sensitivity (98%), specificity (94%) and false detection rate (4%) ( Supplementary File 5). However, one Group A variant was not called and false positives were called at three Group B sites. Deeper evaluation of these results by the companies could help them improve their variant calling performance.

The ICR142 Benchmarker report for each company provides a clear summary of performance overall and for indels and base substitutions separately. The report also gives specific benchmarking information about Group A variants and Group B sites in a simple, clear and concise fashion ( Supplementary File 4 and Supplementary File 5). The report further highlights if missing data prevents assessment at any given site, as was the case for one negative site in the data from Company B ( Supplementary File 5).

## Conclusion

Evaluation, optimisation and benchmarking of performance, including comparison with current widely-used pipelines, is essential for clinical, commercial and academic NGS variant calling applications. We have developed a tool, ICR142 Benchmarker, to achieve these essential requirements in a consistent, reproducible and transparent fashion, using the ICR142 NGS validation dataset. ICR142 Benchmarker returns useful outputs with various levels of detail to allow both broad and deep understanding of variant calling performance. ICR142 Benchmarker can be applied to VCF files generated by any variant caller, allowing intra- and inter-pipeline comparison. ICR142 Benchmarker is also useful in the optimisation of variant calling algorithms and outputs, including regression testing and filter creation. The ICR142 Benchmarker report provides simple, clear and concise summary statements about a pipeline’s performance, including comparison with the performance of widely-used open-source pipelines. Use of the ICR142 NGS validation series and ICR142 Benchmarker can therefore facilitate in-house optimisation and direct comparison of variant calling methods for NGS data.

## Data availability

The FASTQ files for the ICR142 validation series are available from the European Genome-phenome archive (EGA). The accession number is EGAS00001001332.

## Software availability

ICR142 Benchmarker is available at: https://github.com/RahmanTeamDevelopment/ICR142_Benchmarker/releases

Latest source code: https://github.com/RahmanTeamDevelopment/ICR142_Benchmarker

Archived source code as at time of publication: http://doi.org/10.5281/zenodo.1469013 ^9^

Software license: MIT

The full ICR142 Benchmarker documentation is given in Supplementary File 6 and is available at: https://github.com/RahmanTeamDevelopment/ICR142_Benchmarker/

Supporting data files of GATK, OpEx, DeepVariant v0.5.2 and v0.6.1 input and output files have been archived as a single project file on Open Science Framework: http://doi.org/10.17605/OSF.IO/H3ZR9 ^15^ under a CC0 1.0 Universal licence.

## Supplementary material

**Supplementary File 1**. GATK analysis commands

**Supplementary File 2**. Site-specific ICR142 Benchmarker results for GATK, OpEx and DeepVariant

The description of the column headings are given below:

**Sample** – sample name in the ICR142 NGS validation series

**Gene** – HGNC symbol

**SangerCall** – the most 3’ representation annotated with CSN v1.0 ^16^ or “No” if no variant present

**Type** – “bs”, “del”, “ins”, “complex”, or “indel” for base substitutions, simple deletions, simple insertions, complex indels, or negative indel sites, respectively

**Transcript** – the ENST ID from Ensembl v65 used to annotate the Sanger call

**CHR** – chromosome

**EvaluatedPosition** - evaluated hg19 site position, centre of designed amplicon

**POS** – the left-aligned position in hg19 coordinates for variants or “.” if no variant present

**REF** – the reference allele in hg19 for variants or “.” if no variant present

**ALT** – the alternate allele for variants or “.” if no variant present

**SiteID** – site ID in the ICR142 NGS validation series

**Zygosity** – “heterozygous” a variant that is present on only one allele or “homozygous” a variant that is present on both alleles

**OpEx** – “.” if there is a missing genotype, 0 if site is not called by OpEx, 1 if a base substitution is called when Type = “bs”, or integer value X if X indels are called when Type = “del”, “ins”, “complex”, or “indel”

**OpExExactFinalMatch** – “yes” if CHR, POS, REF and ALT all match when SangerCall is not “No” and OpEx > 0, “no” if CHR, POS REF and ALT do not match when SangerCall is not “No” and OpEx > 0 , “.” otherwise

**GATK** – “.” if there is a missing genotype, 0 if site is not called by GATK, 1 if a base substitution is called when Type = “bs”, or integer value X if X indels are called when Type = “del”, “ins”, “complex”, or “indel”

**GATKExactFinalMatch** – “yes” if CHR, POS, REF and ALT all match when SangerCall is not “No” and GATK > 0, “no” if CHR, POS REF and ALT do not match when SangerCall is not “No” and GATK > 0 , “.” otherwise

**DeepVariant** – “.” if there is a missing genotype, 0 if site is not called by DeepVariant, 1 if a base substitution is called when Type = “bs”, or integer value X if X indels are called when Type = “del”, “ins”, “complex”, or “indel”

**DeepVariantExactFinalMatch** – “yes” if CHR, POS, REF and ALT all match when SangerCall is not “No” and DeepVariant > 0, “no” if CHR, POS REF and ALT do not match when SangerCall is not “No” and DeepVariant > 0 , “.” otherwise

**Group** – “A” if SangerCall is not “No” and GATK, OpEx and DeepVariant are all > 0 , “B” if SangerCall is “No” and GATK, OpEx and DeepVariant are all 0, “.” otherwise

**Supplementary File 3.** Variant calling regression testing of DeepVariant v0.5.2 and v0.6.1

The description of the column headings are given below:

**Sample**– sample name in the ICR142 NGS validation series

**Gene**– HGNC symbol

**SangerCall** – the most 3’ representation annotated with CSN v1.0 ^16^ or “No” if no variant present

**Type**– “bs”, “del”, “ins”, “complex”, or “indel” for base substitutions, simple deletions, simple insertions, complex indels, or negative indel sites, respectively

**Transcript** – the ENST ID from Ensembl v65 used to annotate the Sanger call

**CHR** – chromosome

**EvaluatedPosition** - evaluated hg19 site position, centre of designed amplicon

**POS** – the left-aligned position in hg19 coordinates for variants or “.” if no variant present

**REF** – the reference allele in hg19 for variants or “.” if no variant present

**ALT** – the alternate allele for variants or “.” if no variant present

**SiteID** – site ID in the ICR142 NGS validation series

**Group** – “A” if SangerCall is not “No” and GATK, OpEx and DeepVariant are all > 0 , “B” if SangerCall is “No” and GATK, OpEx and DeepVariant are all 0, “.” Otherwise

**DeepVariant v0.5.2** – “.” if there is a missing genotype, 0 if site is not called by DeepVariant v0.5.2, 1 if a base substitution is called when Type = “bs”, or integer value X if X indels are called when Type = “del”, “ins”, “complex”, or “indel”

**DeepVariant v0.6.1** – “.” if there is a missing genotype, 0 if site is not called by DeepVariant v0.6.1, 1 if a base substitution is called when Type = “bs”, or integer value X if X indels are called when Type = “del”, “ins”, “complex”, or “indel”

**ConcordantFinalResult v0.5.2** – “no“ if either SangerCall is “No“ and DeepVariant v0.5.2 is >0 or SangerCall is not “No“ and DeepVariant v0.5.2 is “0“ or “.“, “yes“ if SangerCall and DeepVariant v0.5.2 are concordant

**ConcordantFinalResult v0.6.1** – “no“ if either SangerCall is “No“ and DeepVariant v0.6.1 is >0 or SangerCall is not “No“ and DeepVariant v0.6.1 is “0“ or “.“, “yes“ if SangerCall and DeepVariant v0.6.1 are concordant

**ExactFinalMatch v0.5.2** – “yes” if CHR, POS, REF and ALT all match when SangerCall is not “No” and DeepVariant v0.5.2 > 0, “no” if CHR, POS REF and ALT do not match when SangerCall is not “No” and DeepVariant v0.5.2 > 0 , “.” otherwise

**ExactFinalMatch v0.6.1** – “yes” if CHR, POS, REF and ALT all match when SangerCall is not “No” and DeepVariant v0.6.1 > 0, “no” if CHR, POS REF and ALT do not match when SangerCall is not “No” and DeepVariant v0.6.1 > 0 , “.” otherwise

**Supplementary File 4.** ICR142 Benchmarker Report for Company A.

**Supplementary File 5.** ICR142 Benchmarker Report for Company B.

**Supplementary File 6.** ICR142 Benchmarker v1.0.1 documentation.
