# Supplementary material for: ICR142 Benchmarker: evaluating, optimising and benchmarking variant calling performance using the ICR142 NGS validation series
Source: Wellcome Open Res. 2018 Oct 31;3:108. Originally published 2018 Aug 31. [Version 2] doi: 10.12688/wellcomeopenres.14754.2 (PMC6234721; doi:10.12688/wellcomeopenres.14754.2)
Supplement: Supplementary file 5 [file wellcomeopenres-3-16253-s0004.tgz › 5f2aa7cc-92b1-4c35-8c29-7a2a4a1b894f_Supp_file_5.docx]

**ICR142 VARIANT CALLING ANALYSIS REPORT**

|  | |  | | | |
| --- | --- | --- | --- | --- | --- |
|  | |  | | | |
| Submitter: | | **Company B** | |  |  |
| Results: | | | | | |
| \| **Overall** \| **98% sensitivity,** **94% specificity,** **4% false detection rate** \| \| --- \| --- \| \| **Indels** \| **97% sensitivity,** **95% specificity,** **4% false detection rate** \| \| **Base substitutions** \| **100% sensitivity,** **85% specificity,** **5% false detection rate** \| \| Missed 1 Group A variant(s) \| \| \| False positives at 3 Group B negative site(s) \| \| \| 0 variant(s) and 1 negative site(s) in 1 sample(s) had a missing genotype \| \| | | | | | |
|  | | | | | |
|  | | | | | |
|  |  | |  |  | |
| **Notes:** | | | | | |
| Further information about the dataset and methods is available at www.icr.ac.uk/icr142.  Sensitivity, specificity and false detection rate are evaluated with 416 total variants (123 base substitutions and 293 indels) and 288 total negative sites (41 negative for base substitutions and 247 negative for indels).  Any missing genotypes are treated as no calls.  Detection of all 387 Group A variants and avoidance of false positives at all 261 Group B negative sites is expected. Failure to detect a Group A variant or a false positive at a Group B negative site indicates substandard performance. | | | | | |

Date: 2018-07-24
