# Supplementary material for: ICR142 Benchmarker: evaluating, optimising and benchmarking variant calling performance using the ICR142 NGS validation series
Source: Wellcome Open Res. 2018 Oct 31;3:108. Originally published 2018 Aug 31. [Version 2] doi: 10.12688/wellcomeopenres.14754.2 (PMC6234721; doi:10.12688/wellcomeopenres.14754.2)
Supplement: Supplementary file 6 [file wellcomeopenres-3-16253-s0005.tgz › 2695ea52-db5f-4f9b-8a34-863577a453f1_20180822_SupplementaryFile6.pdf]

README.md

ICR142 Benchmarker

Introduction

`ICR142 Benchmarker` is an easy to use tool assessing variant caller performance using the ICR142 NGS validation series. `ICR142 Benchmarker` reports a series of informative metrics with increasing levels of detail from overall calling performance to per site profiles and a one page report summarising both standalone performance and comparative performance with current widely-used open-sourced pipelines.

Prerequisites

`ICR142 Benchmarker` is available for **Mac/Linux**, implemented in R and requires:

1. R version 3.1.2
2. A capacity to build packages from source (requires gcc and gfortran compilers).

Installation

`ICR142 Benchmarker` implements strict version control over all packages and dependencies used by changing the local default R settings. Any R session launched from the same tool directory will have these settings, therefore it is strongly recommended to install the tool into a new directory.

`ICR142 Benchmarker` can be downloaded from GitHub from here in either `.zip` or `.tar.gz` format.

- unpack the compressed file
- Go to main directory: `cd ICR142_Benchmarker-vXXX`
- Install with: `./setup.sh`
  `setup.sh` downloads and installs all required packages and dependencies, automatically creating a `setup.log` file.

Running ICR142 Benchmarker

Once `ICR142 Benchmarker` has been downloaded and successfully installed, run the following command from the main directory of the tool:
`./ICR142_Benchmarker --input input.txt --method_name name [--output path_to_output_directory]`

Input

- **INPUT** file - path to tab separated input file containing:
  `Header line` with SampleID and Location
  `Data` with:

i. Sample IDs in the ICR142 series
ii. Paths to 142 VCF v4.X files

SampleID	Location
D129031	path/to/D129031.vcf
L81899	path/to/L81899.vcf
...	...

- **METHOD** - one word variant caller identifier (can be delimiter-separated)
- **OUTPUT** - path to exitsting or new folder in which outputs will be created. This argument is **optional**, by default "Output_ICR142_Analysis" folder will be created in the main ICR142_Benchmarker directory.

Output

`ICR142 Benchmarker` generates the following files:

- **Summary.txt** - provides summary performance metrics for the evaluated method, specifically the overall sensitivity, specificity and FDR values and the same three metrics calculated for only base substitutions and only indels.
- **FullResults.txt** - tab separated file containing all of the Sanger validation information from the ICR142 dataset and information on the method's performance at each of the 704 sites.
- **FalsePositives.txt** - relevant lines of the VCF files for false positive variant calls.
- **TruePositives.txt** - relevant lines of the VCF files for true positive variant calls.
- **Report.docx** - Word document providing a clear variant calling analysis report of the method's performance on the ICR142 dataset. Key points from the detailed outputs are highlighted to the user, including information about the method's performance in the context of existing best practice.

Column Headings

i Detailed description of all columns in the .txt files can also be found here

---

File: FullResults.txt

Column_name	Description
Sample	sample name in the ICR142 series
Gene	HGNC symbol
SangerCall	the most 3' representation annotated with CSN
Type	`bs`, `del`, `ins`, `complex` or `indel` for *base substitutions*, *simple deletions*, *simple insertions*, *complex indels*, or *negative indel* sites, respectively
Transcript	the ENST ID from Ensembl v65 used to annotate the Sanger call
CHR	chromosome
EvaluatedPosition	evaluated hg19 site position, centre of designed amplicon
POS	the left-aligned position in hg19 coordinates for variants
REF	the reference allele in hg19 for variants
ALT	the alternative allele in hg19 for variants
Zygosity	`homozygous` or `heterozygous` for variants based on Sanger call
SiteID	numeric ID within the ICR142 series
Group	`A`, `B` or `.` see GroupDescriptions

Column_name	Description
<Method_name>	`.` if there is a missing genotype, `0` if site is not called in the submitted call set, `1` if a base substitution is called when Type = *bs*, or integer value `X` if X indels are called when Type = *del*, *ins*, *complex*, or *indel*
ConcordantFinalResult	`no` if either SangerCall is *No* and method_name is *>0* or SangerCall is not *No* and method_name is *0* or *.*, `yes` if SangerCall and method_name are concordant
ExactFinalMatch	`yes` if *CHR*, *POS*, *REF*, and *ALT* all match when SangerCall is not *No*, `no` if *CHR*, *POS*, *REF*, and *ALT* do not match when SangerCall is not *No*, `.` if there is a missing genotype

GroupDescriptions

Total_number	Group	Description
387	`A`	Detection of all Group `A` variants is expected. Failure to detect a Group `A` variant indicates substandard performance
261	`B`	Avoidance of false positives at all Group `B` negative sites is expected. A false positive at a Group `B` negative site indicates substandard performance.

File: TruePositives.txt

Column_name	Description
CHROM	from submitted VCF file
POS	from submitted VCF file
ID	from submitted VCF file
REF	from submitted VCF file
ALT	from submitted VCF file
QUAL	from submitted VCF file
FILTER	from submitted VCF file
INFO	from submitted VCF file
FORMAT	from submitted VCF file
SAMPLE	from submitted VCF file
SiteID	numeric ID within the ICR142 series
Length	length of variant, `0` for Base substitutions, `>0` for indels

File: FalsePositives.txt

Column_name	Description
CHROM	from submitted VCF file
POS	from submitted VCF file
ID	from submitted VCF file
REF	from submitted VCF file
ALT	from submitted VCF file
QUAL	from submitted VCF file
FILTER	from submitted VCF file
INFO	from submitted VCF file
FORMAT	from submitted VCF file

Column_name	Description
SAMPLE	from submitted VCF file
SiteID	numeric ID within the ICR142 series
Length	length of variant, `0` for Base substitutions, `>0` for indels

Notes

VCF Files

- The `VCF` files must each represent a **single sample**.
- ALT column should contain only **one call** (no multi-allelic calls accepted).
- Any base substitution calls are expected to have REF and ALT values of **length one**.

```diff
- incorrect: REF / ALT of GTCA / ATCA
+ correct: REF / ALT of G / A
```

- `Multi-sample VCF` or `gVCF` files should be parsed to fulfill the above criteria.

Data Access and Reproducibility

To allow reproducibility we provide inputs and outputs generated for GATK, OpEx and DeepVariant. Data can be downloaded from OSF cloud.

Links

- Raw data on EGA (Europian Genome Archive)
- OSF
- TGMI

License

Code released under the MIT License.
