# Supplementary material for: ICR142 Benchmarker: evaluating, optimising and benchmarking variant calling performance using the ICR142 NGS validation series
Source: Wellcome Open Res. 2018 Oct 31;3:108. Originally published 2018 Aug 31. [Version 2] doi: 10.12688/wellcomeopenres.14754.2 (PMC6234721; doi:10.12688/wellcomeopenres.14754.2)
Supplement: Peer review history

# Peer review: Referee response for version 2

**DOI:** 10.21956/wellcomeopenres.16253.r34217

**Published:** 2018-11-09

**Authors:** Birgit Sikkema-Raddatz, Lennart F. Johansson

**Affiliations:** 1 Department of Genetics, University of Groningen, University Medical Center Groningen, Groningen, The Netherlands

Thank you for the second version. All points are discussed. Together with the comments it will enable to further value the benchmarker and allow readers to use the benchmarker for several comparisons.

We have read this submission. We believe that we have an appropriate level of expertise to confirm that it is of an acceptable scientific standard.

# Peer review: Referee response for version 2

**DOI:** 10.21956/wellcomeopenres.16253.r34143

**Published:** 2018-11-01

**Authors:** Oliver Hofmann

**Affiliations:** 1 Centre for Cancer Research, University of Melbourne, Melbourne, Vic, Australia

Thank you for the quick response. All my (minor) comments have been addressed. Brad Chapman also has additional resources related to ICR142 available at https://github.com/bcbio/icr142-validation which may be of use to other researchers.

I have read this submission. I believe that I have an appropriate level of expertise to confirm that it is of an acceptable scientific standard.

# Peer review: Referee response for version 1

**DOI:** 10.21956/wellcomeopenres.16077.r34022

**Published:** 2018-10-12

**Authors:** Birgit Sikkema-Raddatz, Lennart F. Johansson

**Affiliations:** 1 Department of Genetics, University of Groningen, University Medical Center Groningen, Groningen, The Netherlands

The manuscript presented by Ruark et al. describes the ICR142 tool that can be used to calculated sensitivity, specificity and false detection rates of NGS variant callers. Making such open source tools available to the community is very valuable. In our opinion the overall quality of the manuscript is good, however, the claims made in the title are too strong.

**Major points:**

1. **Table 1 and in general:  **It should be specified that the benchmarker ICR142 is only for Illumina data, enriched with a TruSeq kit.
  It is mentioned that the values are calculated using the ICR142 dataset. However, the use of the terms sensitivity and specificity imply that these numbers are generalizable to the entire exome. However, the sites are selected using data Illumina TruSeq Exome enrichment procedure in combination with the Illumina HiSeq 2000 sequencing. Difficult positions could differ for different enrichment procedures or sequencing platforms, even between Illumina machines. A variant caller may for instance have a good performance on an Illumina dataset but less on an IonTorrent dataset. 
2. **The title** suggests that variant calling is optimised. However, the manuscript does not discuss optimization of variant calling. It only states that it can be done. In my opinion this is not enough to warrant the statement in the title.
  It would be interesting to further discuss reasons for lower sensitivity and specificity. Only in the Deep Variant tool settings were changed. A more systematic approach for all the tools would be useful. Are there clusters of (types of) variants being missed or called together at different settings. Such a discussion could warrant the optimisation claim.
3. It would be interesting to discuss how the benchmarking results for one or more of the aligner/variant calling combinations compares to other benchmarking datasets. For instance on high-confidence variants in a Genome In A Bottle dataset.

 

**Minor points**

**The title** suggests that variant calling in general is evaluated. However, only germline SNV and Indel variants are taken into account. Copy number variant calls or somatic/mosaic variants are not benchmarked. The types of variants evaluated should be specified more clearly.

**Page 3:**

**Introduction:** we agree with the comment of reviewer 1 that evaluation of published pipelines is not limited.

**Method:** The selection criteria of the 704 variant sites can be stated more clearly.

**Figure 1** states that there are 288 Sanger validated negative sites. This is further specified in 41 sites without a base substitution and 247 sites without an indel. This suggests that the 41 sites do have an indel and the 247 sites do have a SNV. Is this the case or are the negative sites negative for both indels and variants. If so, specify.

**Table 1 **should include the used aligner as well as the variant caller in the column headers. As is stated correctly in the methods and results section, the variant callers aren’t isolated units and the aligner could influence the result (although the results show this is not the case here). A general remark: the data are not easy to read and understand.

**Page 5**: “regression testing” should be explained, also how to perform.

**Page 5/6: **It would be interesting to discuss if the 4% FDR in companies A and B and DeepVariant are constituted by the same variants/regions and how this compares to the 2% and 5% of OpEx and GATK.

**Page 6: **It is stated that the information of Companies A and B is shown in table 1. This is not the case.

**Page 6:** utility in creation of variant detection filters:

 The filter setting to improve performance was tested for DeepVariant. What about the other caller?

 A more systematic approach is required.

 We could not locate the 3 Vcf files to reproduce the output.

We have read this submission. We believe that we have an appropriate level of expertise to confirm that it is of an acceptable scientific standard, however we have significant reservations, as outlined above.

# Response

**Published:** 2018-10-25

**Authors:** Nazneen Rahman

**Affiliations:** The Institute of Cancer Research and The Royal Marsden NHS Foundation Trust, UK

**General Response**

 We thank the reviewers for their comments. We believe they may have a slight misunderstanding of our intentions in writing this paper and in making ICR142 Benchmarker. In 2016 we made the ICR142 dataset available (Ruark et al10.12688/f1000research.8219.2). We did this simply because we had been using the dataset in-house and had found it useful and we thought others might also find it useful. Many have - the paper has been read >1000x and downloaded >200x. Following discussions and feedback with ICR142 users we made ICR142 Benchmarker. We believe/hope it makes using the ICR142 dataset easier and more useful.

 We have included some simple use cases in this paper to highlight potential uses, but naturally there will be situations where ICR142 and hence ICR142 Benchmarker will not be suitable.

**Response to reviewers comments:**

**Major points**

- **Table 1 and in general:  **It should be specified that the benchmarker ICR142 is only for Illumina data, enriched with a TruSeq kit. It is mentioned that the values are calculated using the ICR142 dataset. However, the use of the terms sensitivity and specificity imply that these numbers are generalizable to the entire exome. However, the sites are selected using data Illumina TruSeq Exome enrichment procedure in combination with the Illumina HiSeq 2000 sequencing. Difficult positions could differ for different enrichment procedures or sequencing platforms, even between Illumina machines. A variant caller may for instance have a good performance on an Illumina dataset but less on an IonTorrent dataset.

Response: Thank you. We have added to the abstract, paper and Table 1 legend that these are Illumina data. In the paper, we have added ‘The exome sequence data was generated using the Illumina TruSeq Exome and a HiSeq2000 sequencer. Full details of the ICR142 series are given in Ruark et al’. ICR142 data can, and has, been used with data from other capture kits. All the ICR142 sites have been orthogonally validated so the results are not dependent on the enrichment process or sequencer. Indeed we believe that the ICR142 dataset and ICR142 Benchmarker have particular utility in helping to uncover this type of performance variability.

- **The title** suggests that variant calling is optimised. However, the manuscript does not discuss optimization of variant calling. It only states that it can be done. In my opinion this is not enough to warrant the statement in the title.

Response: Thank you. We had inadvertently omitted the word ‘performance’ from the title, which we have now added.

- It would be interesting to further discuss reasons for lower sensitivity and specificity. Only in the Deep Variant tool settings were changed. A more systematic approach for all the tools would be useful. Are there clusters of (types of) variants being missed or called together at different settings. Such a discussion could warrant the optimisation claim.

Response: Thank you. These are exactly the types of questions we hope people will find ICR142 Benchmarker useful in highlighting, evaluating and optimizing. We have extensively used ICR142 dataset in the optimization of OpEx, as described in the OpEx paper.

- It would be interesting to discuss how the benchmarking results for one or more of the aligner/variant calling combinations compares to other benchmarking datasets. For instance on high-confidence variants in a Genome In A Bottle dataset.

Response: Thank you. We agree these types of comparisons would be interesting. We hope that people will be inclined to do them, and as more people use (and hopefully make available) their ICR142 Benchmarker outputs we believe there may be several opportunities for interesting comparisons.

**Minor points**

- **The title** suggests that variant calling in general is evaluated. However, only germline SNV and Indel variants are taken into account. Copy number variant calls or somatic/mosaic variants are not benchmarked. The types of variants evaluated should be specified more clearly.

Response: Thank you. This is important. We have added to the abstract the following. ‘Here we present ICR142 Benchmarker, a tool to generate outputs for assessing germline base substitution and indel calling performance using the ICR142 NGS validation series’

**Page 3: **

- **Introduction:** we agree with the comment of reviewer 1 that evaluation of published pipelines is not limited.

Response: We agree with you both. We have changed this sentence to. ‘Evaluations of pipeline performance are often based on internal data. This makes comparison, standardisation and regulation of NGS variant calling performance difficult.’

- **Method:** The selection criteria of the 704 variant sites can be stated more clearly.

Response: Details for this are given in the original ICR142 publication. We have included in the revised paper ‘Full details of the ICR142 series are given in Ruark et al’.  

- **Figure 1** states that there are 288 Sanger validated negative sites. This is further specified in 41 sites without a base substitution and 247 sites without an indel. This suggests that the 41 sites do have an indel and the 247 sites do have a SNV. Is this the case or are the negative sites negative for both indels and variants. If so, specify.

Response: Each site was inspected and negative or positive for the specified type of variation. No other assumptions should be made. i.e. it should not be assumed that the 41 sites without a base substitution have an indel.

- **Table 1 **should include the used aligner as well as the variant caller in the column headers. As is stated correctly in the methods and results section, the variant callers aren’t isolated units and the aligner could influence the result (although the results show this is not the case here). A general remark: the data are not easy to read and understand.

Response: Thank you for this suggestion. We have included this.

- **Page 5**: “regression testing” should be explained, also how to perform.

Response: We have explained the term in the paper as follows: “Regression testing ensures previously developed and tested pipelines still perform in the same way after updates have been implemented.” We don’t feel it is appropriate to state how it should be performed, because there are many different ways to do regression testing, dependent on many factors. The aim of our paper is simply to provide a dataset and tool that people might find useful when doing regression testing.

- **Page 5/6: **It would be interesting to discuss if the 4% FDR in companies A and B and DeepVariant are constituted by the same variants/regions and how this compares to the 2% and 5% of OpEx and GATK.

Response: We agree that these types of comparisons would be interesting. We didn’t have permission from Companies A and B to use their data in this way.

- **Page 6: **It is stated that the information of Companies A and B is shown in table 1. This is not the case

Response. Thank you for bringing this to our attention. We have removed this for Companies A and B.

- **Page 6:** utility in creation of variant detection filters:The filter setting to improve performance was tested for DeepVariant. What about the other caller? A more systematic approach is required.

Response: Thank you. In line with the recommendations for software tool articles we have included representative ‘use cases’. The filter setting for DeepVariant is an example of one of these use cases. It was not our aim to perform a systematic approach to filter setting. Though we would be delighted if the ICR142 dataset and ICR142 Benchmarker were used in this way.

- We could not locate the 3 Vcf files to reproduce the output.

Response: We have checked and all files are there and available to download. Of note, there are not three VCF files but rather 3 zipped files containing 142 VCF files each.

# Peer review: Referee response for version 1

**DOI:** 10.21956/wellcomeopenres.16077.r33804

**Published:** 2018-09-24

**Authors:** Oliver Hofmann

**Affiliations:** 1 Centre for Cancer Research, University of Melbourne, Melbourne, Vic, Australia

Ruark *et al* present 'ICR142 Benchmarker', a tool to simplify comparing locally generated SNV calls against a Sanger-validated benchmark set using the previously published ICR142 sample set available from EGA. Additional methods to assess variant callers are always needed, and the easy software installation, pre-configured comparisons and result summaries make this task more accessible to groups without dedicated bioinformatics support. The tool is freely available under the MIT license and well-documented. 

 ## Major comments

- I would not agree that the evaluation of published pipelines is limited in the literature; I've lost count of the number of papers assessing variant callers, exome capture methods and other aspects of HTS workflows. That said, ICR142 Benchmarker simplifies the comparison for researchers which is worth emphasizing.
- Readers might also benefit from a *brief* comparison to other available benchmarking datasets (Genome in a Bottle, COLO829, ...); the ICR142 set benefits from the breadth of having a large number of different samples, but doesn't cover as much of the genome as some of the WGS-based evaluation sets.
- Likewise, how does ICR142 Benchmarker compare to existing frameworks such as RTG's vcfeval or Illumina's hap.py/som.py? Again, I see the use case for Benchmarker but the target audience might not.
- I am not sure I quite understand how InDels are handled. Does Benchmarker compare just the *size* of the insertion/deletion (at a given position)? Are InDels left-aligned as part of the VCF parsing step, or is this task left to the user?

## Minor comments

- A brief description of the ~700 covered site would be helpful to understand what ICF142 does/does not cover. Do any variants overlap difficult to sequence regions (e.g., from Heng Li's paper ^1^) or other low complexity regions? 
- It is worth mentioning that the ICR142 set covers germline variant calls from human lymphocyte DNA (apologies if I missed this being pointed out somewhere).
- Based on the GitHub repository it looks like the assessment is limited to hg19. Could benchmark data for GRChr37 and, more importantly, GRCh38 be made available? If not, worth mentioning in the paper.
- Could DeepVariant's low base substitution specificity be due to working with Stampy-generated alignments rather than bwa-mem alignment? I realize the assessment of callers is not the focus of this paper, but this might still be worth testing. 
- It might be helpful to have a sample (Word) report in the GitHub repository.

I have read this submission. I believe that I have an appropriate level of expertise to confirm that it is of an acceptable scientific standard, however I have significant reservations, as outlined above.

## References

[1] LiH: Toward better understanding of artifacts in variant calling from high-coverage samples. *Bioinformatics*.2014;30(20) : 10.1093/bioinformatics/btu356 2843-51 10.1093/bioinformatics/btu356 24974202PMC4271055

# Response

**Published:** 2018-10-25

**Authors:** Nazneen Rahman

**Affiliations:** The Institute of Cancer Research and The Royal Marsden NHS Foundation Trust, UK

## Major comments

- I would not agree that the evaluation of published pipelines is limited in the literature; I've lost count of the number of papers assessing variant callers, exome capture methods and other aspects of HTS workflows. That said, ICR142 Benchmarker simplifies the comparison for researchers which is worth emphasizing.

Response: We agree with you. We have changed this sentence to ‘Evaluations of pipeline performance are often based on internal data. This makes comparison, standardisation and regulation of NGS variant calling performance difficult.’

- Readers might also benefit from a *brief* comparison to other available benchmarking datasets (Genome in a Bottle, COLO829, ...); the ICR142 set benefits from the breadth of having a large number of different samples, but doesn't cover as much of the genome as some of the WGS-based evaluation sets. Likewise, how does ICR142 Benchmarker compare to existing frameworks such as RTG's vcfeval or Illumina's hap.py/som.py? Again, I see the use case for Benchmarker but the target audience might not.

Response: We made ICR142 Benchmarker available following interactions with users of the ICR142 dataset, to enhance its usability and potential utility. We are pleased you can see possible advantages compared with other datasets / frameworks and we would be delighted if someone wanted to evaluate this more formally, but as you say that wasn’t the purpose of this paper. Overall, we personally favour using as many as possible – one almost always gains something from such evaluations.  

- I am not sure I quite understand how InDels are handled. Does Benchmarker compare just the *size* of the insertion/deletion (at a given position)? Are InDels left-aligned as part of the VCF parsing step, or is this task left to the user?

Response: We considered detection and annotation/representation of indels separately, because there remains considerable variation in how the same indel can be described. If an indel was called within a 200bp window centred on the site position we considered an indel to have been detected and it is counted as a Positive indel call. It is possible that the annotation/representation of the indel might differ. The indels are not left-aligned by ICR142 benchmarker. 

## Minor comments

- A brief description of the ~700 covered site would be helpful to understand what ICR142 does/does not cover. Do any variants overlap difficult to sequence regions (e.g., from Heng Li's paper ^1^) or other low complexity regions? 

Response: We have included in the revised paper ‘Full details of the ICR142 series are given in Ruark et al’. The original paper describes how we selected the 704 sites. 13 of the sites (SiteIDs: 150, 169, 191, 221, 235, 473, 502, 512, 523, 534, 543, 580, 639) overlap those in Heng Li’s paper.

- It is worth mentioning that the ICR142 set covers germline variant calls from human lymphocyte DNA (apologies if I missed this being pointed out somewhere).

Response: Thank you. Yes it is. We have added ‘for assessing germline base substitution and indel calling performance….’ To the abstract.

- Based on the GitHub repository it looks like the assessment is limited to hg19. Could benchmark data for GRChr37 and, more importantly, GRCh38 be made available? If not, worth mentioning in the paper.

Response: Thank you. Great point. We have updated ICR142 Benchmarker so this is possible. In the paper we have added ‘It can be used with hg19/GRCh37 or hg38/GRCh38 data.’

- Could DeepVariant's low base substitution specificity be due to working with Stampy-generated alignments rather than bwa-mem alignment? I realize the assessment of callers is not the focus of this paper, but this might still be worth testing. 

Response: Thank you. This is exactly the type of question we hoped ICR142 Benchmarker might stimulate, though it is not a focus of our work to take it any further. In this paper we simply wanted to highlight some possible use cases, to give an idea of how people might find the ICR142 series and ICR12 Benchmarker useful.  

- It might be helpful to have a sample (Word) report in the GitHub repository.

Response: Thank you. We have updated the GitHub repository to include a sample (Word) report.
